# Collateral circulation caused by end-stage hepatic *alveolar echinococcosis*

**DOI:** 10.1186/s12879-022-07970-7

**Published:** 2023-05-15

**Authors:** Tiemin Jiang, Tuerganaili Aji, Bo Ran, Qiang Guo, Ruiqing Zhang, Ayifuhan Ahan, Abuduaini Abulizi, Talaiti Tuergan, Yingmei Shao, Hao Wen

**Affiliations:** 1 State Key Laboratory of Pathogenesis, Prevention and Management of High Incidence Diseases in Central Asia, The First Clinical Medical College of Xinjiang Medical University, Urumqi, China; 2 Department of Hepatobiliary and Hydatid Diseases, Digestive and Vascular Surgery Center, The First Affiliated Hospital of Xinjiang Medical University, Urumqi, 830054 China; 3 The First Clinical Medical College of Xinjiang Medical University, Urumqi, 830054 China; 4 Xinjiang Clinical Research Center for Echinococcosis and Hepatobiliary Diseases, First Affiliated Hospital of Xinjiang Medical University, Urumqi, China

**Keywords:** Collateral circulation, Hepatic *alveolar echinococcosis*, End-stage, Portal vein cavernous degeneration

## Abstract

**Background:**

Hepatic *alveolar echinococcosis *(HAE), as a benign parasitic disease with malignant infiltrative activity, grows slowly in the liver, allowing sufficient time for collateral vessels to emerge in the process of vascular occlusion.

**Methods:**

The portal vein (PV), hepatic vein and hepatic artery were observed by enhanced CT and the inferior vena cava (IVC) by angiography, respectively. Analysis of the anatomical characteristics of the collateral vessels helped to look into the pattern and characteristics of vascular collateralization caused by this specific etiology.

**Results:**

33, 5, 12 and 1 patients were included in the formation of collateral vessels in PV, hepatic vein, IVC and hepatic artery, respectively. PV collateral vessels were divided into two categories according to different pathways: type I: portal -portal venous pathway (13 cases) and type II: type I incorporates a portal-systemic circulation pathway (20 cases). Hepatic vein (HV) collateral vessels fell into short hepatic veins. The patients with IVC collateral presented with both vertebral and lumbar venous varices. Hepatic artery collateral vessels emanating from the celiac trunk maintains blood supply to the healthy side of the liver.

**Conclusions:**

Due to its special biological nature, HAE exhibited unique collateral vessels that were rarely seen in other diseases. An in-depth study would be of great help to improve our understanding related to the process of collateral vessel formation due to intrahepatic lesions and its comorbidity, in addition to providing new ideas for the surgical treatment of end-stage HAE.

## Introduction

Collateral circulation refers to the opening of the communicating branches between blood vessels when the normal vascular pathway is seriously narrowed or blocked, which alleviates the high pressure and achieves self-compensation. This plays a vital role in the blood supply and reflux of patients' liver to ensure liver function.

AE is confined to the northern hemisphere, especially restricted in such areas as central and Eastern Europe, Russia, China, northern Japan and northern region of North America [1]. It proliferates in an invasive manner, constantly produces new vesicles and penetrates into tissues, similarly as malignancy[2]. It can not only directly invade adjacent tissues, but also metastasize via lymphatic and blood supplies to retroperitoneal and distant organs, such as the brain and lungs [3, 4]. Its slow growth in the liver leads to chronic vascular occlusion, which provides sufficient time for compensatory collateral angiogenesis, with unique clinical features exhibited. When the arteries, portal vein (PV), hepatic vein (HV) and Inferior vena cava (IVC)were severely invaded, the corresponding collateral circulation may occur to maintain liver circulation and function. Not only collateral circulations arising from the particular disease but also the compensatory capacity of the hepatic vascular system was a big shock to us.

Unfortunately, except case report, there have been few reports on hepatic collateral circulation due to this particular pathogenesis. As a leading medical institution in areas with a high prevalence of *echinococcosis*, our center has been dedicated to its diagnosis and treatment for many years and accumulated much experience. This study explored the collateral circulation of each vascular system in liver with end-stage hepatic AE, which may help to improve our understanding of the process and complications of collateral angiogenesis caused by intrahepatic lesions and provide new ideas for surgical treatment.

## Patients and methods

This retrospective case series included 51 end-stage HAE patients with collateral circulation diagnosed and treated at the digestive vascular surgery center, the First Affiliated Hospital of Xinjiang Medical University between January 2010 and December 2021. The study was approved by the ethics committee of the First Affiliated Hospital of Xinjiang Medical University; (K202206-10). Due to the retrospective nature of the study, the committee waived the requirement for individual consent. Enhanced CT was performed to examine the PV, hepatic vein, and hepatic artery while Angiography was performed to examine the IVC. The results were evaluated jointly by two senior radiologists.

The inclusion criteria were (1) patients with a definite diagnosis of HAE (2) patients whose CT or DSA showed that the PV, IVC, hepatic vein or hepatic artery was seriously invaded by the lesion to cause blockage and exhibited obvious collateral vessels (3) patients were conscious enough to complete all examinations. The exclusion criteria were patients (1) with other liver diseases or severe comorbidities (such as cirrhosis, liver dysfunction due to persistent obstructive jaundice) (2) incomplete important clinical data.

33, 5, 12 and 1 patients were included in the formation of collateral vessels in PV, hepatic vein, IVC and hepatic artery, respectively. PV collateral vessels were divided into two categories according to different pathways: type I: portal venous-portal venous pathway and type II: type I incorporates a portal-systemic circulation pathway, based on which we studied the anatomical characteristics of the collateral circulation of these vessels and compared the leukocytes, hemoglobin, platelets and liver function of these patients.

### Statistical methods

The statistical software IBM SPSS 26.0 (IBM Corp, Armonk, NY, USA) was used for data analysis. Normally distributed continuous variables were expressed as Means ± SD and analyzed by t-test; abnormally distributed continuous variables were expressed as median and analyzed by Mann–Whitney u-test. p < 0.05 was considered a statistically significant difference.

## Results

**Fig. 1:**
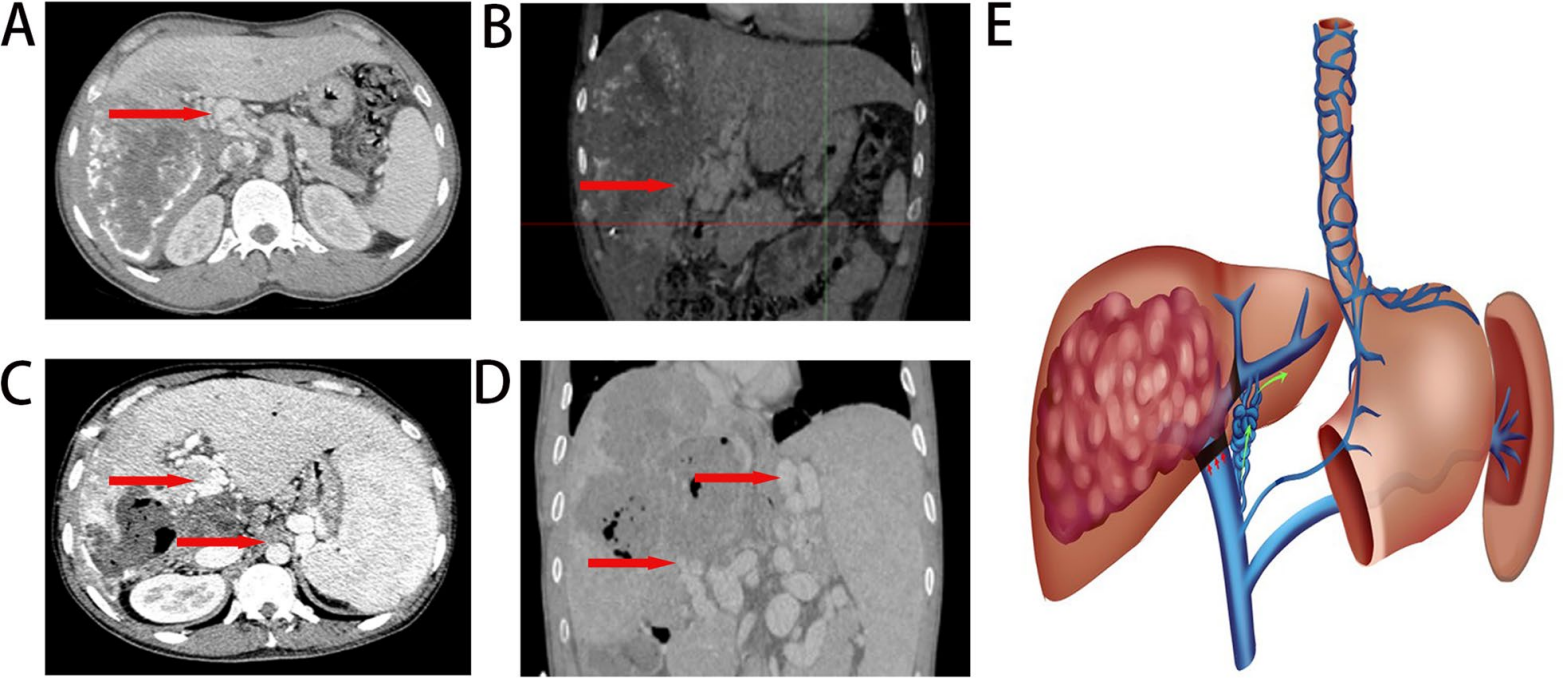
portal vein collateral vessels: **A**, **B** collateral vessels type I: Portal—portal venous pathway. **C**, **D** Type II: type I incorporates a portal-systemic circulation pathway. **E** Schematic representation of the portal collateral circulation

**Fig. 2:**
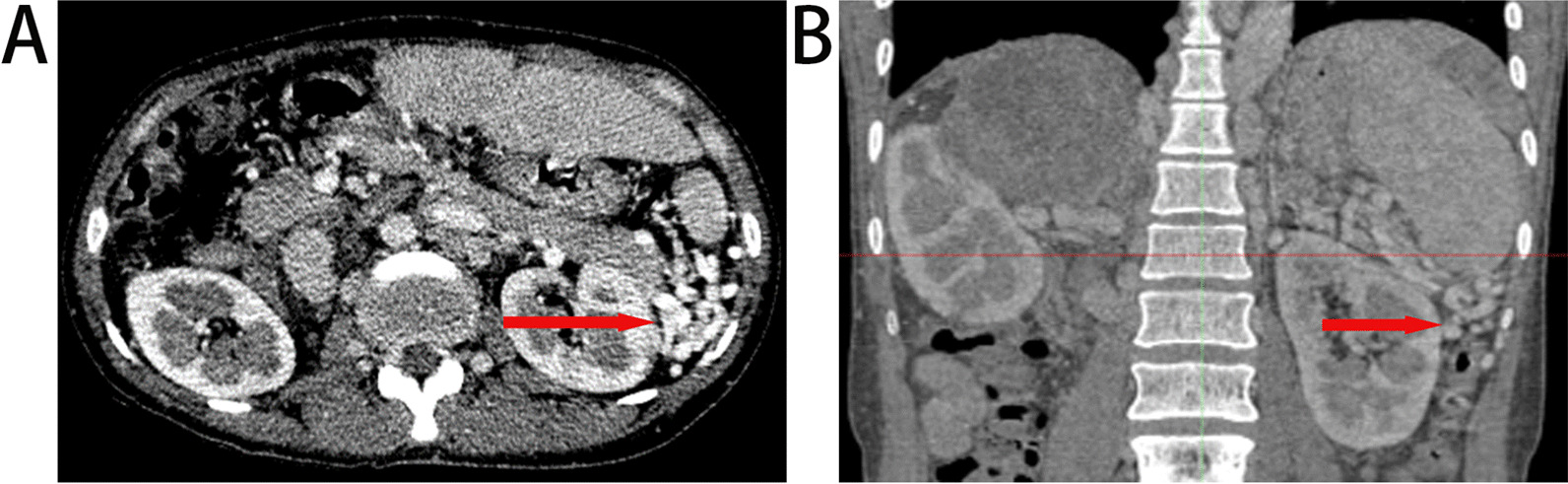
portal collateral vessels: varicose vessels located in the lower pole of the spleen: **A** Transverse CT image of the portal collateral vessels located in the lower pole of the spleen. **B** Coronary CT image of the portal collateral vessels located in the lower pole of the spleen

The 33 patients with PV collateral vessels were divided into two categories according to the different pathways: Type I: portal-portal venous pathway (13 cases) and type II: type I combined with PV- Systemic circulation pathway (20 cases). Abdominal distention and abdominal pain were listed among the most prominent symptoms (18). 12 patients suffered obstructive jaundice due to invasion of bile ducts by hydatid lesions (5 in type I,7 in type II); and three had previous gastrointestinal bleeding (all type II). Among the 20 patients with type II, due to the opening of the systemic circulation, varices of the esophagogastric fundic veins were observed in 15 cases, splenomegaly in 20 cases, and ascites in 3 cases (Table 1). In patients with two types of portal collateral circulation, the platelet count was significantly lower in type II patients than that in type I (P < 0.05). There were no significant differences between the two groups in leukocytes, hemoglobin, portal transpeptidase, ALT, total bilirubin, direct bilirubin, indirect bilirubin and alkaline phosphatase (P > 0.05) (Table 2).In addition, two patients presented with varicose vessels in the lower pole of the spleen, which were considered to be mesenteric veins ( Figs. 1,2).

**Table 1:** Clinic data of portal vein Cavernous degeneration patients

	Type I	Type II	Total
Total cases	13	20	33
Gender			
Male	8	11	19
Female	5	9	14
Age	38(21–57)	39.5(17–66)	39(17–66)
Ethnicity			
Tibet	7	11	18
Kazakh	1	3	4
Han	1	5	6
Uygur	2	0	2
Kyrgyz	1	0	1
Mongol	1	1	2
Hui	0	0	0
Previous surgical history			
NO	5	11	16
PTCD	1	2	3
PVE	0	0	0
ALPPS	0	0	0
ERCP	0	0	0
Hepatectomy	5	4	9
Albendazole administration			
Number of patients	1	1	2
Duration (month)	36	1	
Child–Pugh classification			
A	5	7	12
B	8	12	20
C	0	1	1
Extrahepatic lesions			
Lung	2	1	3
Brain	0	0	0
Kidney	2	0	2
Bone (sacrum, patella)	0	0	0
Symptoms			
Abdominal pain	6	12	18
Gastrointestinal bleeding	0	3	3
Jaundice	6	6	12
Physical examination	4	3	7
Physical signs			
Esophagogastric fundic varices	0	15	15
Splenomegaly	0	20	20
Ascites	0	3	3

**Table 2:** The comparison of blood routine and liver function in two groups

Variable	Type I	Type II	p
PLT	187.92 ± 79.78	133.68 ± 57.12	< 0.05
HGB	115.08 ± 20.50	101.26 ± 19.64	> 0.05
WBC	5.85 ± 1.57	5.03 ± 1.58	> 0.05
TB	28.02 ± 26.19	32.67 ± 20.72	> 0.05
DB	10.46 ± 15.56	9.35 ± 11.14	> 0.05
IBIL	12.69 ± 11.15	17.52 ± 14.29	> 0.05
ALT	42.04 ± 32.89	47.47 ± 58.59	> 0.05
AST	41.69 ± 20.06	50.56 ± 38.34	> 0.05
ALP	321.70 ± 285.02	306.75 ± 262.98	> 0.05

The collateral vessels of the hepatic vein fell into short hepatic veins (5 cases). 2 patients showed obstruction of the main trunk of the hepatic vein followed by dilatation of the communicating branch of the hepatic vein, with blood flowing through the short hepatic vein into the IVC via the traffic branch.

All 12 patients with collateral angiogenesis presented with both vertebral and lumbar venous varices. 10 of the 12 patients had no obvious symptoms; one had mild abdominal pain; another one had jaundice due to bile duct invasion. All patients had no significant lower limb edema or ascites.

Collateral vessels of the hepatic artery (1 case) maintained the blood supply to the healthy side of the liver in the form of an abdominal aorta—left hepatic artery.

## Discussion

When the important vascular trunk of the liver is blocked for some reason, collateral vessels may appear to compensate for the function of blood return, most commonly seen in tumors and acute thrombosis. Due to the unique biological characteristics of HAE, we observed that this disease can also have their own characteristics and interesting collateral vessels, which are described as follows:

### PV

In case of PV obstruction, there can be two collateral vascular pathways. one is the portal vein-portal vein pathway, proposed by Balfour and Stewart [5] in 1869, which is known as cavernous degeneration and featured by the formation of tortuous dilated vessels around the first PV, connecting the extrahepatic PV to the intrahepatic PV. The other is the portal—systemic circulation pathway, flowing into the hemi-azygos vein and the superior vena cava [6] through the PV-left esophagogastric vein, esophagogastric fundic plexus and splenic vein and then which is also a common pattern of collateral circulation in portal hypertension caused by cirrhosis.

The HAE patients we observed showed differences in both the incidence and clinical symptoms of PV cavernous degeneration from other diseases. All patients with severe portal trunk occlusion developed cavernous degeneration, with a much higher incidence than reported in thrombosis [6]. 18 patients among the 33 collateral vessels of PV suffered abdominal distension and abdominal pain compared with only 3 with gastrointestinal bleeding. The main symptom of collateral formation caused by other diseases reported is upper gastrointestinal hemorrhage, which is significantly higher than our results [7–11]. All of this may be related to the fact that AE invasion of the vessels is a chronic process, allowing enough time to gradually establish type I collateral vessels, which relieves the venous pressure in the main PV. In addition, it is noteworthy that we observed both type I and type II collateral vessel morphology, but a separate portal-somatic circulation collateral morphology was absent. Based on the above observations, we hypothesized that the degree and speed of PV trunk obstruction was closely related to the form of PV collateral branch formation. Narrow and mild PV obstruction would allow its surrounding collateral veins to cross the obstruction site and drain the distal blood to the intrahepatic portal branches; that is, the portal vein-portal vein shunt was first formed to maintain normal portal perfusion. As the extent and degree of PV obstruction increased, the portal-portal blood flow was insufficient to relieve portal hypertension, and some of the blood flow was directed to the lower pressure systemic circulation, forming a portal vein- systemic circulation. Khanna and Shiv K. Sarin also made a similar point [11, 12].

The incidence of portal vein cavernous degeneration after portal trunk invasion is higher in HAE patients than in acute portal vein embolism (57.2%), and completely different from those of patients with cirrhosis whose primary manifestation is the establishment of a pure portal-systemic circulation [13, 14]. Therefore, we speculated that when prehepatic portal hypertension occurred and the liver was in good condition without other diseases, type I portal collateral branches served as the main mode of compensatory blood flow. The slower the portal vein was occluded, the higher the incidence of cavernous deformation would be, which coincided with other reports [6]. Moreover, HAE invaded the PV at a low level, between the I-II branches, and was less likely to be combined with umbilical vein dilation. None of the patients showed dilatation of the umbilical vein or abdominal wall vein.

**Fig. 3:**
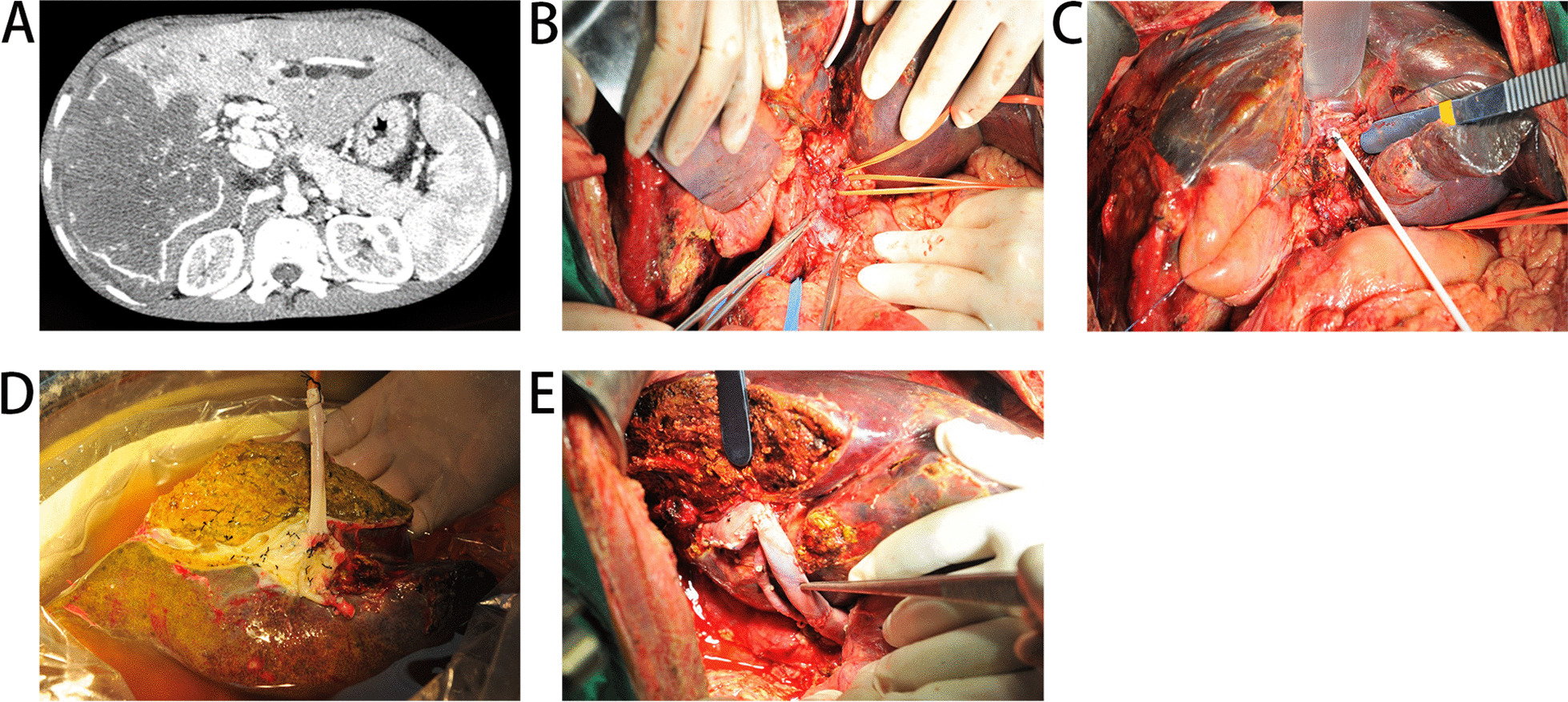
treatment of portal cavernous variant by ELRA: **A** CT image of hepatic vesicular hydatid induced portal cavernous degeneration. **B** Varicose vessels are seen in the hilar area. **C** The round ligament of the liver is catheterized to guarantee extracorporeal perfusion of the liver. **D** reconstruction of the portal vein with blood vessels in patients after cardiac death **E** anastomosis of the portal vein

The treatment of such patients was very tricky and liver resection may risk uncontrollable bleeding. Ex vivo liver resection and autotransplantaion (ELRA) enjoyed good intraoperative bleeding control and materialized radical resection. We performed ELRA treatment for end-stage HAE combined with portal vein cavernous degeneration in 9 patients, with initial good results (Fig. 3), hoping to serve as a new alternative treatment.

### IVC

**Fig. 4:**
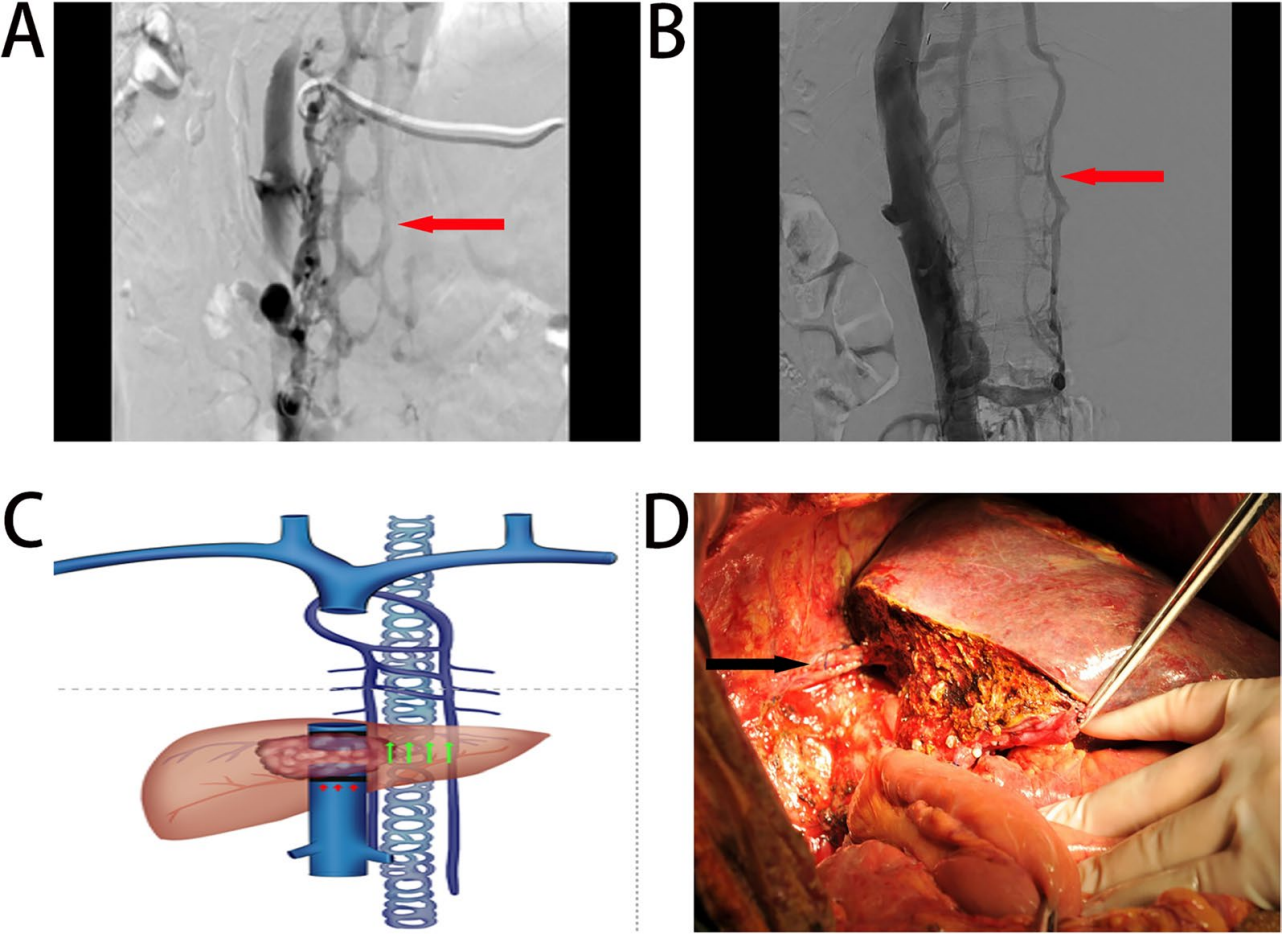
collateral vessels of the inferior vena cava: **A** vertebral venous plexus vessel shown by arrow. **B** Lumbar ascending vein shown by arrow **C** schematic representation of the collateral vessels of the inferior vena cava. **D** No reconstruction of the inferior vena cava. The hepatic vein is anastomosed end-to-end to the superior and inferior vena cava as indicated by the arrow

The Vascular collateralization of the IVC has been reported in details by Kapur [15], but the vertebral and lumbar veins have not been described, which may differ from the specific location of the IVC invaded by HAE. The posterior hepatic segment of IVC between the left atrium and the right renal vein was the main site involved. The collateral circulation of the IVC was thought to have two main pathways: the lumbar venous plexus and the vertebral venous plexus [16] (Fig. 4). Via the four lumbar veins symmetrical to the left and right sides and the lumbar ascending veins, left side of the lumbar venous moves into the hemiazygos vein and the right side enters the superior vena cava and returns to the atrial along the azygos vein. IVC angiography revealed that the collateral vessels of the vertebral venous plexus were dominated by the intraspinal veins, which ascended along the spinal canal. It had been reported to be an important route for communicating the superior and IVC, as well as intra—and extracranial, because of the lack of venous valves and the extensive communicating branches at other venous plexuses (intracranial venous sinus, thoracic, abdominal, pelvic venous plexus), which was of great physiological and clinical importance in the spread of infection, metastasis of tumors, and the formation of collateral circulation [17–19]. One patient out of 12 showed a metastatic lesion in the brain but not in the lung. Is it possible that this is the route by which neglected hepatic AE metastasize to the brain?

Clinically, IVC obstruction was manifested by ascites and lower limb edema. Most patients with HAE are asymptomatic because of the well-established collateral circulation. The presence or absence of significant symptoms served as an important basis for determining whether the collateral circulation was well established after complete IVC obstruction, and played an important role in deciding which surgical approach to reconstruct the IVC. Our previous study proposed a surgical approach without reconstruction of the IVC in the presence of well-established collateral circulation, yielding good results [20, 21]. But for which careful preoperative evaluation and implementation lay the basis.

It was of concern that the collateral circulation of the inferior vena cava was difficult to observe by CT and was thus usually visualized by angiography. Therefore, once the inferior vena cava was severely obstructed, it was recommended to improve the inferior vena cava angiography to well assess whether there was collateral circulation established.

### Hepatic vein

**Fig. 5:**
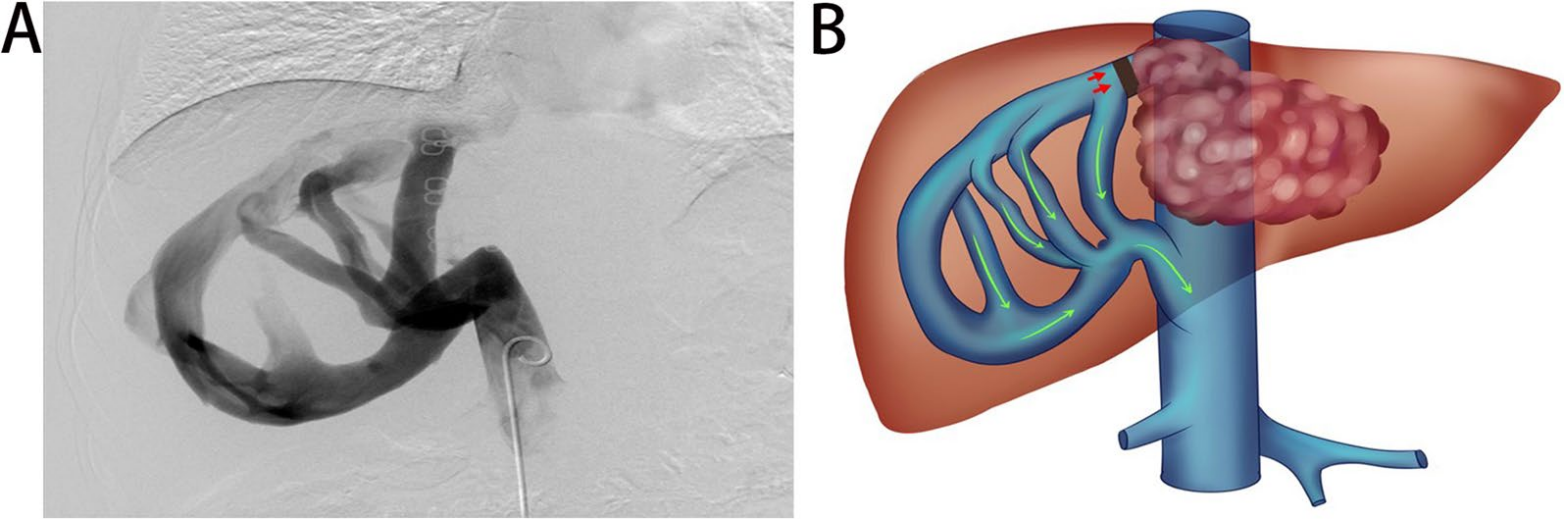
collateral vessels of the intrahepatic veins: **A** Angiography of the collateral vessels of the intrahepatic veins. **B**: Schematic representation of the collateral vessels of the intrahepatic veins

Collateral circulation of the hepatic vein has rarely been reported in other diseases. It is generally accepted that the short hepatic vein is an important compensatory vessel [22]. When the hepatic vein was obstructed, the short hepatic vein became abnormally thick and compensated for the function of the hepatic vein. In two patients, complete obstruction of the hepatic vein was followed by a dilated traffic vessel between the hepatic vein and the short hepatic vein, in which case blood flowed through the traffic branch into the short hepatic vein and then converged into the IVC (Fig. 5). Restricted by a few reports available in the form of case reports [23–25], we hypothesized that the appearance of intrahepatic venous plexus resulted from the original presence of fine intervening venous traffic branches in the liver, which gradually expanded and acted as mutual traffic when the main hepatic venous trunk was obstructed. However, if similar traffic vessels were lacking in the liver, the main hepatic venous reflux area would cause congestion, while the uninvolved short venous return area remained unaffected. This may have important implications for ELRA, since both the main hepatic trunk and the short hepatic veins was to be reconstructed when the liver formed thicker short hepatic veins and the remaining liver tissue remained congested. If there was an intrahepatic venous plexus and obstruction of the main hepatic trunk without obvious signs of liver congestion, it was feasible to reconstruct only the main hepatic trunk and suture the short hepatic vein. One case was reconstructed with ELRA and no significant postoperative complications occurred.

### Hepatic artery

**Fig. 6:**
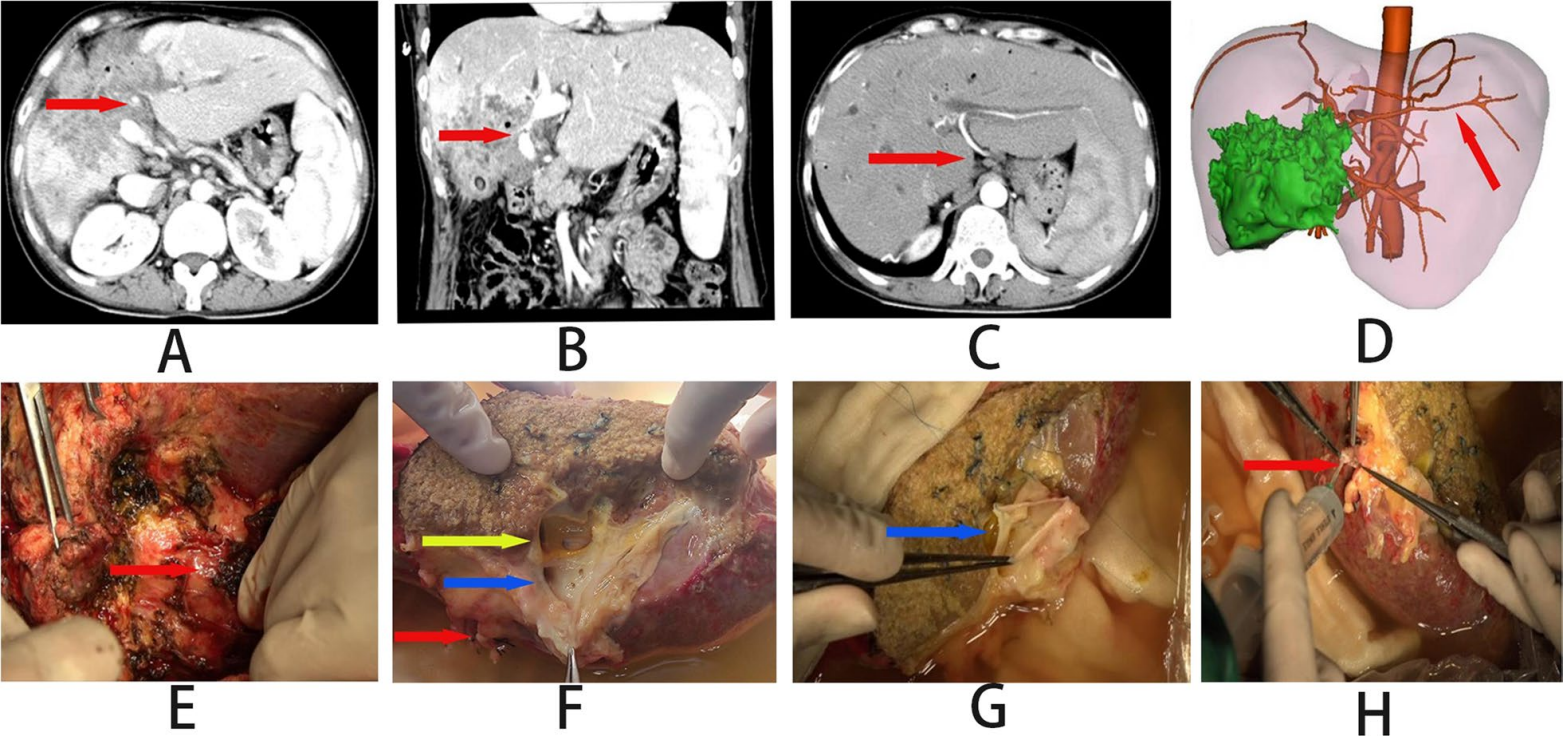
**A**–**D** preoperative imaging findings of the patient: **A** the arrow shows: the main hepatic artery is invaded **B** the main portal vein is invaded; **C** Arrow in **D** collateral vessel of hepatic artery. **E** Intraoperatively, the first hilum is seen to be severely invaded. **F** Red arrow: hepatic artery; Blue arrows: portal vein; Yellow arrows: intrahepatic biliary tract **E** reconstructed portal vein **F** collateral vessels of hepatic artery **G**: the blue arrow shows: portal vein; **H**: the red arrow shows: hepatic artery

The collateral circulation of the hepatic artery in patients with hepatic AE has rarely been reported, but it has been reported in small numbers in patients with HCC because of its particular implications for interventional treatment. We found one case in which the main hepatic artery was invaded, with an artery emanating from the coeliac trunk and running within the hepato-gastric ligament to supply blood to the healthy left liver (Fig. 6). Although it is possible that the artery could be a variant, it does also act as a blood compensation similar to collateral circulation. Phrenic artery is the most common variant of arterial blood supply for liver cancer patient, with equal origin from either the aorta or coeliac axis. It is worth exploring whether direct resection of the originally invaded arterial trunk with reconstruction of only the PV and biliary tract is feasible in patients with end-stage vesicular *echinococcosis* in whom arterial vascular collaterals are present and the first hepatic hilum invaded.

There are also many shortcomings. Due to the invasive nature of venography, not all cases in this study were observed by angiography. In addition, this study was restricted by a small sample size.

## Conclusions

HAE, as a benign tumor-like disease, has its own special biological characteristics and therefore appears with collateral vessels that are difficult to present in other diseases. An in-depth study of it will be of great help to improve our understanding of the process of collateral vessel formation due to intrahepatic lesions and its comorbidity, in addition to providing new ideas for the surgical treatment of end-stage HAE.

## Data Availability

The datasets generated during and/or analysed during the current study are not publicly available due to protect study participant privacy, but are available from the corresponding author on reasonable request.

## Abbreviations

HAE: Hepatic *alveolar Echinococcosis*
HV: Hepatic vein
IVC: Inferior vena cava
PV: Portal vein
ELRA: Ex vivo liver resection and autotransplantaion
